# Associations Between Interoceptive Awareness, Psychological State, and Physical Performance in Sport-Science Students: A Cross-Sectional Study

**DOI:** 10.3390/bs16101784

**Published:** 2026-10-01

**Authors:** Zhaoqing Li, Emir Mert Koçoğlu, Özgür Eken, Tahir Volkan Aslan, Yuhao Su

**Affiliations:** 1 Sports Department, Hunan Women’s University, Changsha 410004, China; lizhaoqing@hnwu.edu.cn; 2 Department of Physical Education and Sports, Institute of Health Sciences, İnönü University, 44280 Malatya, Türkiye; 38251402002@ogr.inonu.edu.tr; 3 Department of Physical Education and Sport Teaching, Faculty of Sport Sciences, İnönü University, 44280 Malatya, Türkiye; 4 Department of Travel, Tourism and Entertainment Services, Erdemli Vocational School, Mersin University, 33000 Mersin, Türkiye; tvolkanaslan@mersin.edu.tr; 5 Faculty of Education, Henan University, Kaifeng 475001, China; yuhaosu@henu.edu.cn

**Keywords:** interoception, perceived stress, anxiety, sleep quality, physical performance, sport-science students, sport psychology, cross-sectional study

## Abstract

Background/Objectives: Interoceptive awareness may support stress regulation in sport, but its association with objective physical performance remains unclear. This study examined relationships between self-reported interoceptive awareness, perceived stress, anxiety, sleep quality, and field-based performance in sport-science students. Methods: Eighty-five students (54 men, 31 women; 20.65 ± 2.43 years) completed the MAIA-2, PSS-10, STAI, and PSQI and performed handgrip, countermovement-jump, sit-and-reach, and bilateral Y-Balance tests. Analyses included Pearson correlations, Welch tests with false-discovery-rate correction, HC3-robust hierarchical regression, and exploratory age- and sex-adjusted bootstrap indirect-effect models. Results: The MAIA-2 composite was inversely associated with perceived stress (r = −0.461, 95% CI [−0.614, −0.275], *p* < 0.001) but showed no detectable association with physical performance (r = −0.003 to 0.136; all q ≥ 0.961); equivalence testing indicated that four of the five performance correlations were statistically equivalent to zero within a ±0.30 bound. Women reported higher stress and anxiety, whereas men showed greater handgrip strength and jump height. Individual-sport participants reported higher state anxiety than team-sport participants (g = −0.886, q = 0.004). Interoceptive awareness did not significantly increase explained variance in any performance model, and exploratory indirect-effect confidence intervals all included zero. Conclusions: Self-reported interoceptive awareness was related to perceived stress but was not detectably related to brief field-based performance in this sample. Longitudinal and experimental studies are needed to test temporal and causal mechanisms.

## 1. Introduction

Interoception refers to the sensing, interpretation, and regulation of signals originating within the body, including cardiovascular, respiratory, gastrointestinal, thermoregulatory, nociceptive, and musculoskeletal information (Craig, 2002; Khalsa et al., 2018). Contemporary accounts emphasize that interoception is not a unitary construct. Self-reported interoceptive awareness or sensibility, behavioral interoceptive accuracy, and metacognitive awareness of interoceptive performance capture partly distinct levels of bodily information processing (Khalsa et al., 2018; Forkmann et al., 2016). The Multidimensional Assessment of Interoceptive Awareness, Version 2 (MAIA-2) was developed to characterize multiple self-reported dimensions of adaptive body awareness rather than objective signal-detection accuracy (Mehling et al., 2012; Mehling et al., 2018). This distinction is especially important when interoception is examined in relation to athletic performance.

Interoceptive processes matter for exercise because athletes must continuously estimate their internal physiological state, regulate effort, and anticipate fatigue. Predictive-processing and allostatic frameworks propose that the brain combines incoming bodily signals with prior expectations, and uses this to allocate physiological resources and guide behaviour (Barrett & Simmons, 2015; Barrett, 2017). In exercise settings, this perspective has been extended to perceived fatigue as a metacognitive inference about deviations from expected bodily state (Dekerle et al., 2025). Consistent with this view, athletes with greater cardiac interoceptive sensitivity regulate freely chosen physical effort more effectively (Herbert et al., 2007), and interoceptive processes appear to shape how sustained exercise and fatigue are perceived (Greenhouse-Tucknott et al., 2022; McMorris, 2020).

Evidence from athletic populations is growing but remains heterogeneous. Elite runners have demonstrated differences from non-athletes in selected self-reported and behavioral dimensions of interoception, particularly under attentional challenge (Seabury et al., 2023). More recently, multidimensional assessment has shown that athletes and non-athletes may differ across interoceptive sensibility, accuracy, and awareness, with effects varying by the dimension assessed (Zeng et al., 2025). These findings do not necessarily imply that a self-report measure such as the MAIA-2 should correlate with every form of physical performance. Short-duration maximal tasks such as handgrip strength and countermovement jumping depend strongly on neuromuscular and anthropometric characteristics, whereas sustained endurance tasks place more explicit demands on effort monitoring and pacing. Establishing where self-reported interoceptive awareness does and does not relate to performance is therefore theoretically informative.

Psychological state may provide one potential link between interoceptive awareness and athletic functioning. Adaptive awareness of bodily signals has been discussed as a resource for emotion regulation and stress management (Farb et al., 2015; Price & Hooven, 2018). Anxiety is also closely connected to the interpretation of bodily signals, although heightened interoceptive attention can be adaptive or maladaptive depending on context and appraisal (Khalsa et al., 2018). Sleep quality is relevant because insufficient or disturbed sleep impairs cognitive and neuromuscular function and slows recovery (Fullagar et al., 2015). Recent sport-specific work has also linked poorer sleep to selected lower-body neuromuscular outcomes (Cabarkapa et al., 2024). These variables plausibly relate to both interoceptive awareness and performance, but few studies have measured them together in athletic samples.

Sex and sport type may further shape psychological and physical outcomes. Biological sex is associated with substantial differences in several determinants of strength and power performance, although individual variation and training history remain important (Ansdell et al., 2020). Psychological profiles may also differ across sport contexts. In some studies, individual-sport athletes reported more anxiety or depressive symptoms than team-sport athletes, possibly because responsibility for the outcome is less shared and social support less immediate (Pluhar et al., 2019; Kemarat et al., 2022). These comparisons are relevant as secondary contextual analyses rather than as evidence of fixed biological or sport-specific causation.

The present study extends this literature in three specific ways. First, whereas Seabury et al. (2023) and Zeng et al. (2025) compared athletes with non-athletes on interoceptive dimensions, neither paired multidimensional self-report with a battery of objective field tests. The present design therefore asks a different question: not whether athletes differ in interoception, but whether interoceptive self-report tracks measurable physical output within an athletic-student population. Second, Herbert et al. (2007) and Greenhouse-Tucknott et al. (2022) examined interoception in tasks requiring sustained effort regulation, whereas the present battery deliberately samples brief maximal and capacity-oriented tasks. This contrast is informative. Predictive-processing accounts (Barrett & Simmons, 2015; Dekerle et al., 2025) predict that interoceptive inference should matter more when effort must be continuously monitored than when a single maximal output is produced.

Third, studies linking body awareness to stress regulation (Farb et al., 2015; Price & Hooven, 2018) and those examining sleep and neuromuscular performance (Fullagar et al., 2015; Cabarkapa et al., 2024) have generally been conducted in separate studies. The present study measured interoceptive awareness, perceived stress, state and trait anxiety, sleep quality, and four physical performance domains in one session. This allows the associations to be compared within a single sample rather than across studies that differ in population and protocol. This design makes it possible to test whether self-reported interoceptive awareness relates selectively to psychological state, to physical performance, or to both.

Accordingly, the primary aim of this study was to quantify the cross-sectional association between self-reported interoceptive awareness and multiple field-based physical performance outcomes in sport-science students. The investigator’s expectation was a positive association between interoceptive awareness and physical performance. Predictive-processing accounts nevertheless allow an alternative possibility: associations may be negligible for short-duration maximal tasks in which neuromuscular and anthropometric factors predominate. Both possibilities are therefore considered when the findings are interpreted. For psychological outcomes, an inverse association with perceived stress was expected. Secondary aims were to examine associations with perceived stress, anxiety, and sleep quality and to compare study variables by sex and sport type. Exploratory cross-sectional indirect-effect models assessed whether perceived stress, trait anxiety, or sleep quality statistically linked interoceptive awareness with physical performance. Because all variables were measured at one time point, these models were not interpreted as evidence of temporal or causal mediation (Maxwell & Cole, 2007).

## 2. Materials and Methods

### 2.1. Study Design and Reporting

This was a single-center, cross-sectional observational study conducted at the Faculty of Sport Sciences, İnönü University, Malatya, Türkiye. The manuscript was prepared in accordance with the Strengthening the Reporting of Observational Studies in Epidemiology (STROBE) recommendations (von Elm et al., 2007). Ethical approval was obtained before recruitment (see Section 2.3), and the study was subsequently registered at ClinicalTrials.gov (NCT07696234). The registration was submitted after data collection had been completed; it is therefore reported as a retrospective study record and not as evidence of a prospectively registered analysis plan. The core correlation and regression analyses were investigator-planned but were not externally preregistered. Equivalence testing, Y-Balance sensitivity analyses, MAIA-2 subscale sensitivity analyses, expanded regression reporting, and the competition-level-adjusted sport-type model were conducted post hoc during peer review to address specific reviewer concerns. The global MAIA-2 composite is therefore described as investigator-defined rather than registry-defined.

### 2.2. Participants and Sample Size

Participants were recruited by convenience sampling from undergraduate students enrolled in the Faculty of Sport Sciences at İnönü University. Eligibility criteria were: (1) age ≥18 years, (2) current undergraduate enrollment in the faculty, (3) ability to complete the physical test battery, and (4) provision of written informed consent. Exclusion criteria were an acute injury or illness likely to affect physical performance on the assessment day, a contraindication to any physical test, or an incomplete assessment battery. No participant who began the assessment was excluded for incomplete data.

An a priori sample-size calculation was performed using G*Power (version 3.1.9.7; Heinrich Heine University Düsseldorf, Düsseldorf, Germany) (Faul et al., 2009). For a multiple-regression framework with six predictors, f^2^ = 0.25, α = 0.05, and 80% power, the minimum required sample was 62. To allow for unusable or incomplete data, recruitment continued beyond this threshold. The final sample comprised 85 participants. This calculation was designed for the multivariable regression framework and should not be interpreted as a dedicated power analysis for small indirect effects. A post hoc sensitivity analysis indicated that, with *N* = 85, α = 0.05 and 80% power, the smallest detectable bivariate correlation was |r| = 0.30 (|r| = 0.34 at 90% power). Associations smaller than this threshold could therefore not be reliably detected. In response to peer review, a post hoc equivalence analysis was added to distinguish failure to detect an association from evidence that effects of at least small-to-moderate magnitude were unlikely (Section 2.8).

### 2.3. Ethics and Data-Collection Procedure

The protocol was approved by the İnönü University Health Sciences Scientific Research Ethics Committee (Decision No. 2026/9991; Session 8; 21 April 2026). Data were collected from 11 to 17 May 2026. All participants received written and verbal information about the study, potential risks, confidentiality, and the voluntary nature of participation and provided written informed consent before assessment. The study was conducted in accordance with the principles of the Declaration of Helsinki.

Each participant completed a single assessment session lasting approximately 45 min. Participants completed the session in a fixed order so that physical exertion could not influence their questionnaire responses: informed consent, demographic and sport-history questionnaire, self-report instruments, standardized warm-up, then physical performance testing. The physical tests were completed in the same order for all participants: handgrip strength, countermovement jump, sit-and-reach flexibility, and Y-Balance testing. Adequate recovery was provided between attempts as detailed below. The fixed test order is acknowledged as a limitation because order effects cannot be excluded.

### 2.4. Demographic and Sport Characteristics

Participants reported age, sex, height, body mass, sport discipline, active/license status, years of sport experience, training sessions per week, training duration per day, and competition level. The training-frequency variable refers to weekly training sessions rather than calendar days; one swimmer reported eight weekly sessions because of twice-daily training. Competition level was recorded as İl (local/provincial), Bölge (regional), or Ulusal (national); it was available for 74 of the 76 participants included in sport-type comparisons. Body mass index (BMI) was calculated as kg/m^2^. Sport type was classified from the reported discipline: football, volleyball, handball, and basketball were categorized as team sports; boxing, taekwondo, badminton, fitness, athletics, swimming, kickboxing, tennis, karate, and gymnastics were categorized as individual activities. Nine participants reported no active sport and were excluded only from sport-type comparisons. All other analyses used the full sample of 85.

### 2.5. Interoceptive Awareness

Self-reported interoceptive awareness was assessed with the 37-item MAIA-2 (Mehling et al., 2018), which includes eight canonical subscales: Noticing, Not-Distracting, Not-Worrying, Attention Regulation, Emotional Awareness, Self-Regulation, Body Listening, and Trusting. Items are rated from 0 (never) to 5 (always). The Turkish version has published psychometric data (Özpınar et al., 2021). The canonical MAIA-2 interpretation is subscale-based. For the investigator-planned global-level analyses, an operational 37-item composite was calculated as the mean of all appropriately keyed items after reversing items 5–10, 11, 12, and 15. The resulting composite ranges from 0 to 5, with higher scores indicating greater overall self-reported interoceptive awareness. Because this operational composite is not a canonical validated unidimensional total score, it is referred to throughout as the “MAIA-2 composite.” To address the reviewer’s construct-validity concern directly, post hoc sensitivity analyses were additionally performed using the eight canonical subscales; these analyses are reported separately and do not redefine the primary variable.

### 2.6. Perceived Stress, Anxiety, and Sleep Quality

Perceived stress was measured using the 10-item Perceived Stress Scale (PSS-10), which assesses the degree to which situations in the previous month were appraised as unpredictable, uncontrollable, and stressful (Cohen et al., 1983). Items are rated on a five-point scale; positively worded items are reverse-scored before summation. The Turkish adaptation has demonstrated acceptable psychometric properties (Eskin et al., 2013). Higher scores indicate greater perceived stress.

State and trait anxiety were measured using the 40-item State-Trait Anxiety Inventory (STAI) (Spielberger et al., 1970), comprising separate 20-item state and trait scales. The Turkish adaptation by Öner and Le Compte (1985) was used. Higher scores indicate greater state or trait anxiety.

Sleep quality was assessed with the Pittsburgh Sleep Quality Index (PSQI), a 19-item self-report instrument that yields seven component scores and a global score ranging from 0 to 21 (Buysse et al., 1989). Higher global scores indicate poorer sleep quality; scores >5 are commonly interpreted as indicating poor sleep. The validated Turkish version was used (Ağargün et al., 1996). Because the PSQI global score intentionally aggregates heterogeneous sleep dimensions, internal-consistency alpha was not used as the primary reliability index for this composite.

### 2.7. Physical Performance Tests

The test battery was selected to sample the physical qualities most commonly profiled in sport-science curricula and in applied field settings: maximal isometric strength, lower-limb explosive power, posterior-chain flexibility, and dynamic postural control. All four are portable, require no laboratory instrumentation, and can be completed by a full class cohort within a single timetabled session, which was a practical requirement of the present design. We acknowledge, however, that this pragmatic rationale does not align optimally with the theoretical account motivating the study. Predictive-processing models implicate interoception chiefly in the continuous monitoring and regulation of effort, and the tasks used here are completed within seconds and are constrained largely by neuromuscular and anthropometric capacity. Endurance, pacing, fatigue-regulation, or decision-making-under-strain protocols would provide a more direct test of that account, but they require individual laboratory sessions, standardized pre-test control, and sustained participant motivation that were not feasible in this setting. The present battery therefore tests whether self-reported interoceptive awareness generalizes to the performance qualities routinely screened in this population, rather than whether interoception supports effort regulation as such. This boundary is stated explicitly in Section 4.1 and Section 4.8.

Handgrip strength was assessed using a digital hand dynamometer (Baseline^®^ Model 12-0286, Fabrication Enterprises Inc., White Plains, NY, USA). Participants stood with the tested arm alongside the body and the elbow fully extended and performed three maximal contractions with the dominant hand. Thirty seconds of recovery separated attempts, and the highest value (kg) was analyzed.

Countermovement-jump (CMJ) height was assessed using the My Jump 2 smartphone application on an iPhone (Apple Inc., Cupertino, CA, USA). Smartphone-based flight-time analysis has demonstrated high agreement with force-platform measures of jump height (Balsalobre-Fernández et al., 2015). Participants kept their hands on their hips and performed three maximal countermovement jumps without a pause at the transition from downward to upward movement. Sixty seconds separated attempts, and the highest jump height (cm) was analyzed.

Flexibility was assessed using a standard sit-and-reach box. Participants sat barefoot with the knees fully extended and the soles of the feet against the box and reached forward maximally with both hands. Three trials were completed with 30 s of rest, and the highest reach distance (cm) was retained.

Dynamic balance was assessed bilaterally using the Y-Balance Test, which is derived from the Star Excursion Balance Test and has established measurement reliability when standardized procedures are followed (Plisky et al., 2009). Participants maintained single-leg stance while reaching maximally in the anterior, posteromedial, and posterolateral directions. Reach distances were normalized to limb length, and a composite score (%) was calculated separately for the right and left stance legs.

Testing conditions were held as constant as the setting allowed rather than experimentally controlled. All participants completed the battery in the same fixed order, in the same facility, with the same examiners and equipment, and after an identical standardized warm-up, and all questionnaires were completed before any physical exertion. Beyond these measures, familiarization trials, caffeine and food intake, time of day, recent training load, and menstrual-cycle phase were neither standardized nor recorded. Data collection was embedded in scheduled class hours across a single week, which fixed the testing window and precluded repeat visits, individual time-of-day assignment, or pre-test dietary control. These uncontrolled sources of variance are expected to add error to the performance measures and therefore to reduce, rather than inflate, observed correlations; they are revisited in Section 4.7.

### 2.8. Statistical Analysis

Statistical analyses were performed using IBM SPSS Statistics (version 26.0; IBM Corp., Armonk, NY, USA); indi-rect-effect models were estimated with the PROCESS macro for SPSS (version 4.2; Hayes, 2022). Continuous data are reported as mean ± standard deviation (SD) with range where appropriate, and categorical data as n (%). Scale reliability was summarized with Cronbach’s alpha for the MAIA-2 operational composite and subscales, PSS-10, and STAI scales. Primary associations between the MAIA-2 composite and each of the five physical performance outcomes were assessed using Pearson correlation coefficients with 95% confidence intervals (CIs). Associations between the MAIA-2 composite and the four psychological variables were treated as secondary. Benjamini–Hochberg false-discovery-rate (FDR) adjustment was applied within the five physical correlations and within the four psychological correlations (Benjamini & Hochberg, 1995). Because non-significant results cannot by themselves establish the absence of an association, post hoc two one-sided tests (TOST) of equivalence were additionally conducted during peer review for the five MAIA-2–performance correlations. Symmetric equivalence bounds of ±0.30 were used pragmatically because |r| = 0.30 was the smallest bivariate correlation detectable with approximately 80% power in the present sample. A significant TOST result (*p* < 0.05) indicates that the observed association is statistically distinguishable from an effect of at least this magnitude. To examine whether conclusions depended on the non-canonical composite, post hoc construct-sensitivity analyses correlated each of the eight canonical MAIA-2 subscales with the five physical outcomes (40 tests; FDR adjusted across the 40-test family) and with PSS-10 (8 tests; FDR adjusted across the eight-test family).

Sex and sport-type comparisons were evaluated using Welch’s independent-samples *t* test, which does not assume equal group variances. Effect magnitude was expressed as Hedges g and mean difference with 95% CI. To limit multiplicity, FDR correction was applied separately to the 10 sex comparisons and the 10 sport-type comparisons. Adjusted q < 0.05 was considered statistically significant within these families.

For each physical performance outcome, a hierarchical linear regression model was fitted. Step 1 included age and sex (men = 0, women = 1); Step 2 added the MAIA-2 composite; Step 3 added PSS-10, trait anxiety, and PSQI simultaneously. Model contribution was evaluated using R^2^ and change in R^2^ (ΔR^2^). Final-model coefficient inference used HC3 heteroscedasticity-robust standard errors and 95% CIs. A sensitivity model added body mass and years of sport experience to test whether the findings for interoceptive awareness held after adjusting for basic anthropometry and training history. Full final-model coefficients are reported in Supplementary Table S1. The expanded sensitivity models that additionally included body mass and sport experience are reported in full in Supplementary Table S2.

Exploratory parallel indirect-effect models estimated the statistical indirect association between the MAIA-2 composite and each performance outcome through PSS-10, trait anxiety, and PSQI, adjusting for age and sex. Indirect effects were estimated with 5000 bootstrap samples and 95% percentile CIs using standard regression-based procedures (Hayes, 2022). These analyses were explicitly considered cross-sectional association models rather than tests of causal mediation because temporal ordering cannot be established from simultaneous measurements (Maxwell & Cole, 2007). Statistical significance was set at two-sided *p* < 0.05 for the planned model tests, with FDR-adjusted q values used for the multiple-comparison families described above.

## 3. Results

### 3.1. Participant Characteristics and Scale Reliability

All 85 participants completed the study (54 men, 63.5%; 31 women, 36.5%). The mean age was 20.65 ± 2.43 years. Forty-four participants (51.8%) reported a team sport, 32 (37.6%) an individual activity, and nine (10.6%) no active sport. The mean operational MAIA-2 composite was 3.05 ± 0.51. Descriptive statistics are shown in Table 1.

Cronbach’s alpha was 0.847 for the 37-item MAIA-2 operational composite, 0.761 for PSS-10, 0.922 for STAI-State, and 0.849 for STAI-Trait. MAIA-2 subscale alpha values were 0.773 (Noticing), 0.524 (Not-Distracting), 0.381 (Not-Worrying), 0.847 (Attention Regulation), 0.707 (Emotional Awareness), 0.818 (Self-Regulation), 0.836 (Body Listening), and 0.857 (Trusting). Two subscales showed low internal consistency, consistent with reported instability of selected MAIA-2 subscales across samples (Rogowska et al., 2023). We note explicitly that low subscale reliability does not by itself justify greater weight on a composite score: a composite computed from items with weak subscale coherence inherits that weakness rather than resolving it. The operational composite is therefore not presented as a psychometrically superior index, but as a single summary variable used for investigator-planned global-level analyses, with subscale-level analyses reported alongside it (Section 3.2) so that conclusions can be evaluated at both levels. Regarding the scoring scheme, the Turkish adaptation study (Özpınar et al., 2021) reported a six-factor solution in its sample, whereas the present study administered the full 37-item Turkish translation and scored it according to the original eight-subscale structure specified by Mehling et al. (2018). This choice was made for comparability with the international literature, in which the eight-subscale scheme predominates. Because the factor structure of the Turkish version has not been consistently replicated, neither the eight-subscale scoring nor the operational composite should be treated as a validated measurement model in this population; both are reported descriptively.

### 3.2. Associations Between Interoceptive Awareness and Study Outcomes

The MAIA-2 composite was moderately inversely associated with perceived stress (r = −0.461, 95% CI [−0.614, −0.275], *p* < 0.001, q < 0.001). Associations with state anxiety, trait anxiety, and PSQI did not remain statistically significant after FDR correction. No detectable association emerged between the MAIA-2 composite and any of the five physical performance outcomes. Correlation coefficients ranged from approximately zero for handgrip and left Y-Balance to 0.136 for right Y-Balance, and all FDR-adjusted q values were ≥0.961 (Table 2). Post hoc equivalence testing was used to distinguish failure to detect an association from evidence against effects of at least the specified magnitude. The symmetric bounds of ±0.30 were set by the statistical sensitivity of the present sample rather than by any established threshold of practical importance, so the results should be read only within those limits. Using these bounds, correlations were statistically equivalent to zero for handgrip strength (TOST *p* = 0.003), countermovement-jump height (*p* = 0.018), sit-and-reach (*p* = 0.012), and left Y-Balance (*p* = 0.003). Right Y-Balance did not reach equivalence (TOST *p* = 0.059), so a small positive association cannot be excluded for that outcome. These results indicate that associations of |r| ≥ 0.30 are unlikely for four outcomes; they do not exclude smaller associations, and they apply to the operational composite rather than to every MAIA-2 dimension. Full equivalence-test results with 90% CIs are provided in Supplementary Table S4.

Post hoc MAIA-2 subscale sensitivity analyses were added to address the multidimensional structure of the instrument. Across the 40 subscale–performance correlations, four associations were nominally significant before multiplicity correction: Not-Distracting with handgrip strength (r = −0.272, *p* = 0.012), Noticing with sit-and-reach (r = 0.252, *p* = 0.020) and right Y-Balance (r = 0.237, *p* = 0.029), and Emotional Awareness with right Y-Balance (r = 0.220, *p* = 0.043). None survived FDR correction across the 40-test family (all q ≥ 0.385; Supplementary Table S5). In contrast, the inverse relationship with perceived stress was evident across several canonical dimensions: Attention Regulation (r = −0.557, q < 0.001), Trusting (r = −0.440, q < 0.001), Self-Regulation (r = −0.274, q = 0.030), and Noticing (r = −0.247, q = 0.045) remained significant after FDR correction (Supplementary Table S6). Thus, the composite findings should not be interpreted as evidence that all MAIA-2 dimensions behave uniformly.

Because seven participants had a normalized Y-Balance composite above 130% on at least one limb, a sensitivity analysis was conducted. All composite scores were recomputed from the raw anterior, posteromedial and posterolateral reach distances and limb lengths and agreed with the analyzed values to within 0.05 percentage points. Limb-length-to-height ratios ranged from 0.477 to 0.580, consistent with the recorded anterior-superior-iliac-spine to medial-malleolus measurement. In the full sample, the MAIA-2 correlations were r = 0.136 for right Y-Balance and r = −0.003 for left Y-Balance. Excluding the single highest value separately for each limb yielded r = 0.154 (*p* = 0.163) for the right limb and r = −0.021 (*p* = 0.848) for the left limb. Excluding all seven participants with a composite above 130% on either limb (*n* = 78) yielded r = 0.182 (*p* = 0.111) and r = 0.002 (*p* = 0.985), respectively. The interpretation was therefore not driven by the upper-end Y-Balance values.

**Figure 1:**
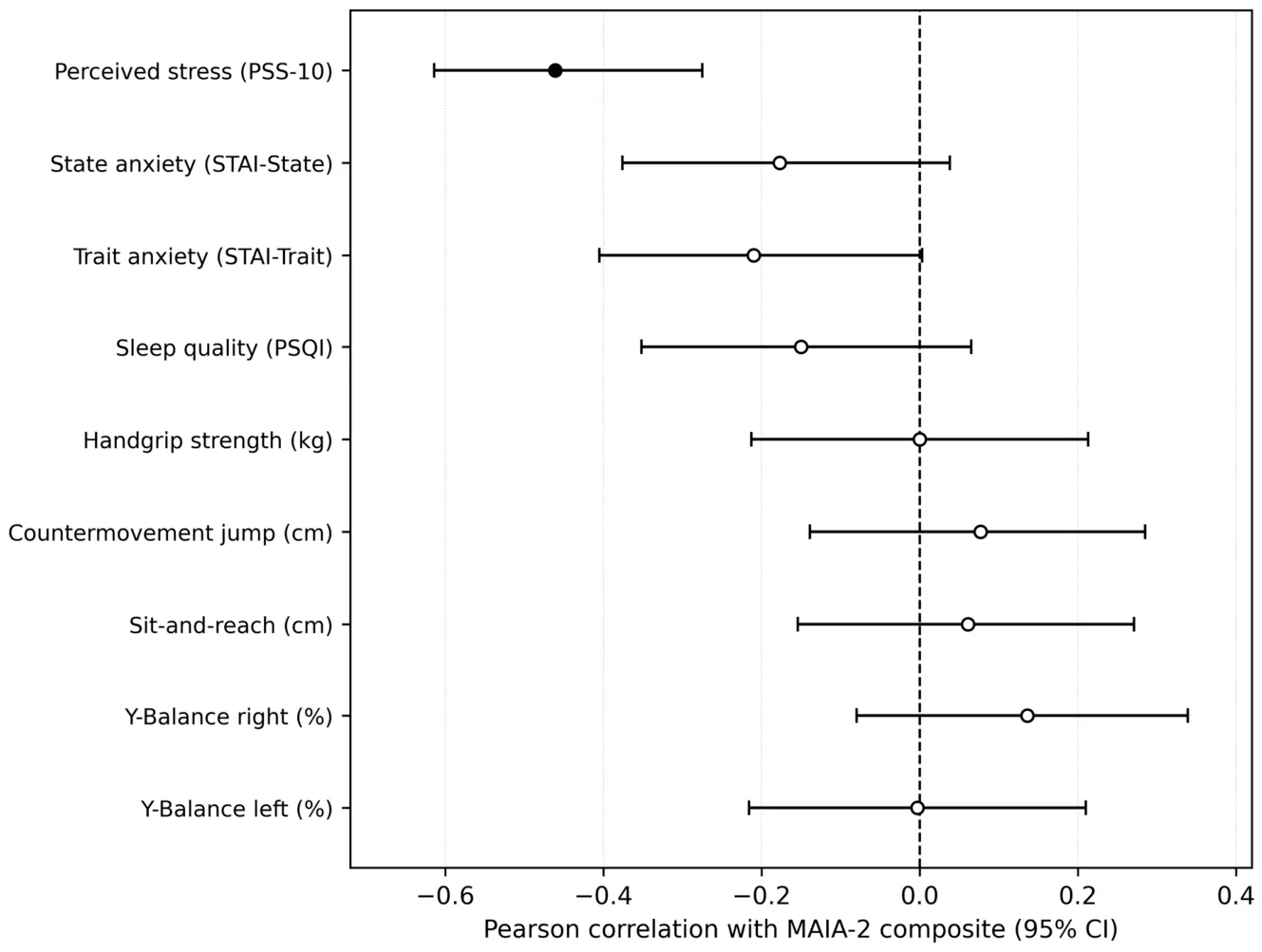
Forest plot of Pearson correlations between the MAIA-2 composite and the psychological and physical outcomes. Filled markers indicate associations that remained significant after false-discovery-rate correction.

Figure 1 visualizes the complete correlation profile. Only one association survived FDR correction: the inverse relationship between the MAIA-2 composite and perceived stress. Its point estimate sits well away from zero, and its 95% CI is comparatively narrow. By contrast, all five physical-performance correlations clustered near zero and their confidence intervals crossed the null. Self-reported interoceptive awareness showed no detectable linear association with brief field-based performance in this sample. The anxiety and sleep estimates were directionally negative but did not survive multiplicity correction.

### 3.3. Sex Differences

After FDR correction, women reported higher perceived stress, state anxiety, and trait anxiety than men, whereas men demonstrated greater handgrip strength and CMJ height (Table 3). The largest standardized differences were observed for handgrip strength (g = 2.306) and CMJ height (g = 1.687). There was no evidence of a sex difference in the MAIA-2 composite, PSQI, flexibility, or bilateral Y-Balance scores. These comparisons describe associations with reported sex and should not be interpreted as isolating biological mechanisms, because body composition and training characteristics were not comprehensively measured.

### 3.4. Sport-Type Differences

Sport-type comparisons included 76 participants with an active sport classification. Individual-sport participants reported higher state anxiety than team-sport participants (mean difference = −9.73 points, 95% CI [−14.94, −4.53], g = −0.886, q = 0.004). Trait anxiety showed an unadjusted difference (*p* = 0.038) but did not survive FDR correction (q = 0.191). No other psychological or physical variable differed after correction (Table 4). Because sport-type groups could differ in composition, the state-anxiety contrast was re-examined with covariate adjustment. The sex distribution did not differ between team- and individual-sport participants (Yates-corrected χ^2^(1) = 1.56, *p* = 0.211). Competition level was available for 74 of the 76 active-sport participants. In a complete-case HC3-robust model adjusting simultaneously for age, sex, years of sport experience, weekly training sessions, and competition level (Bölge and Ulusal indicators with İl as reference), individual-sport participation remained associated with higher state anxiety (B = 9.47 points, 95% CI [4.08, 14.85], *p* < 0.001). Competitive proximity and perceived social support were not measured and remain potential sources of residual confounding.

**Figure 2:**
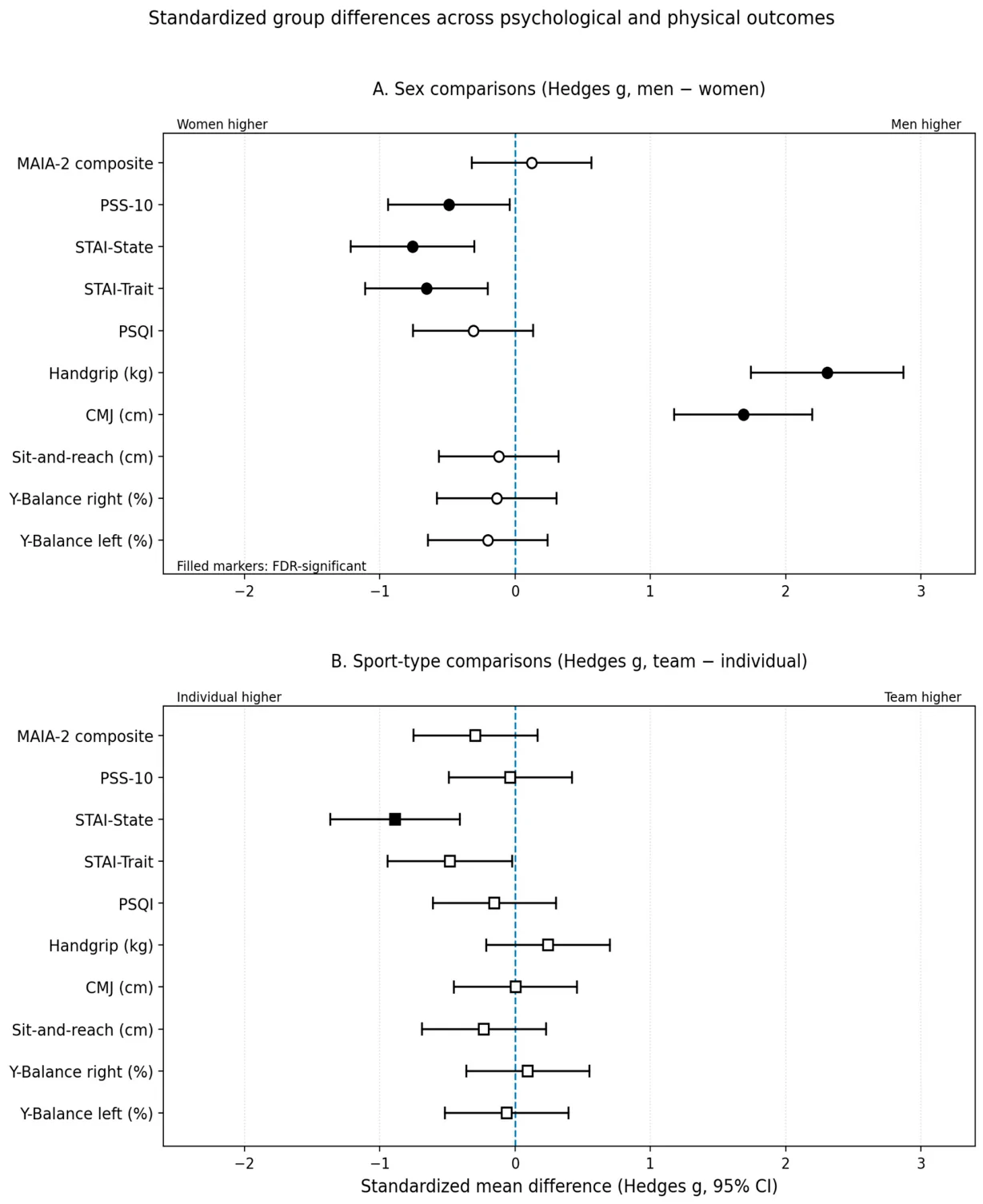
Standardized mean differences (Hedges g) for (**A**) sex (men − women) and (**B**) sport-type (team − individual) comparisons across psychological and physical outcomes. Filled markers indicate differences that remained significant after false-discovery-rate correction.

Figure 2 integrates the standardized group comparisons reported in Table 3 and Table 4. For sex, the largest effects favored men for handgrip strength and countermovement-jump height, whereas women had higher perceived stress and state and trait anxiety; each of these contrasts remained significant after FDR correction. For sport type, the only comparison that remained significant after correction was state anxiety, with individual-sport participants reporting higher scores than team-sport participants. Among the remaining sport-type contrasts, the unadjusted interval for trait anxiety excluded zero (mean difference = −3.77, 95% CI [−7.33, −0.21]) but did not survive FDR correction (q = 0.191); all other intervals crossed zero, indicating substantial uncertainty around those contrasts.

### 3.5. Hierarchical Regression Analyses

Age and sex explained substantial variance in handgrip strength (R^2^ = 0.562) and CMJ height (R^2^ = 0.414), largely because reported sex was strongly associated with these outcomes. Adding the MAIA-2 composite at Step 2 did not significantly increase explained variance for any outcome (ΔR^2^ = 0.000–0.019; all *p* ≥ 0.208). Adding PSS-10, trait anxiety, and PSQI at Step 3 also did not significantly improve any model (all block-change *p* ≥ 0.064). In the final HC3-robust Step 3 models, the MAIA-2 composite was not independently associated with any performance measure (Table 5; complete coefficients in Supplementary Table S1).

PSQI showed an inverse coefficient for CMJ height in the final model (B = −0.676 cm per PSQI point, 95% CI [−1.34, −0.01], *p* = 0.046), but the three-variable psychological block did not significantly increase R^2^ (*p* = 0.064). When body mass and sport experience were added in the expanded sensitivity model, the PSQI coefficient was attenuated (B = −0.642, 95% CI [−1.32, 0.04], *p* = 0.064), whereas the MAIA-2 composite remained non-significant for all five outcomes (all *p* ≥ 0.170). Full coefficients for these sensitivity models are provided in Supplementary Table S2. The sleep-CMJ coefficient was therefore treated as exploratory rather than confirmatory.

As a secondary exploratory analysis, age- and sex-adjusted parallel indirect-effect models were estimated for each performance outcome through perceived stress, trait anxiety, and sleep quality (5000 bootstrap samples). All 15 bootstrap 95% confidence intervals included zero, providing no evidence of a cross-sectional indirect association. Because exposure, mediators, and outcomes were measured simultaneously, these estimates cannot support or refute causal mediation and are reported in full in Supplementary Table S3 and Supplementary Figure S1.

## 4. Discussion

The principal finding of this study was a clear dissociation between psychological and field-performance correlates of self-reported interoceptive awareness. The MAIA-2 operational composite was moderately associated with lower perceived stress, yet it showed no detectable association with handgrip strength, countermovement-jump height, flexibility, or bilateral dynamic balance. This null pattern persisted in hierarchical regression models and in sensitivity analyses incorporating body mass and sport experience. Exploratory indirect-effect models likewise did not provide evidence that perceived stress, trait anxiety, or sleep quality statistically linked interoceptive awareness with physical performance. Secondary analyses identified higher stress and anxiety scores among women, substantially higher grip strength and jump height among men, and higher state anxiety among individual-sport participants after multiplicity correction.

### 4.1. Self-Reported Interoception and Physical Performance

The absence of a detectable direct association between the MAIA-2 composite and the physical test battery should be interpreted cautiously rather than as proof that interoception is irrelevant to performance. A central issue is construct alignment. The MAIA-2 measures self-reported tendencies in attending to, interpreting, and regulating bodily experience (Mehling et al., 2018), whereas behavioral interoceptive accuracy reflects performance on tasks designed to detect or discriminate bodily signals and may correspond only modestly with self-report measures (Forkmann et al., 2016). The post hoc subscale analyses reinforce this distinction: several nominal subscale–performance associations were observed, but none survived correction across the 40-test family. Consequently, the present data support a limited statement about this self-report instrument and these brief field tests, not a general null conclusion about interoception.

A second issue is task demand. Predictive-processing accounts imply that interoceptive information may be particularly consequential when individuals must continuously regulate effort, interpret fatigue, or adapt behavior under uncertainty (Barrett & Simmons, 2015; Dekerle et al., 2025). Herbert et al. (2007), for example, linked interoceptive sensitivity with self-regulation of freely selected physical load, and Greenhouse-Tucknott et al. (2022) examined interoception in a sustained physical-activity context in which fatigue and effort perception were integral to task execution. In contrast, handgrip strength and CMJ height are brief maximal outputs in which force-generating capacity, neuromuscular activation, body size, and training history are likely to dominate. Sit-and-reach and Y-Balance similarly capture flexibility and dynamic control rather than sustained effort regulation. One possible interpretation is that the relevance of interoception to sport performance is task-specific; however, the present design cannot test this directly because no sustained-effort task was included.

This pattern is consistent with evidence that athlete-related differences vary across distinct interoceptive dimensions (Seabury et al., 2023; Zeng et al., 2025), and argues against treating interoception as a single trait expected to predict strength, power, flexibility, and balance uniformly.

### 4.2. Interoceptive Awareness and Perceived Stress

The most robust psychological result was the inverse association between the MAIA-2 composite and perceived stress (r = −0.461). This finding is consistent with theoretical models in which adaptive body awareness contributes to emotion regulation and more flexible responses to physiological arousal (Farb et al., 2015; Price & Hooven, 2018). However, directionality cannot be determined. Three readings remain possible. Greater adaptive interoceptive awareness may help people regulate stress; lower chronic stress may make it easier to attend to bodily signals adaptively; or unmeasured traits such as mindfulness, emotion-regulation ability, or mental health may drive both.

Common-method variance also warrants caution. Interoceptive awareness and perceived stress were measured through self-report during the same session, which can inflate associations through shared response tendencies or contextual influences (Podsakoff et al., 2003). The MAIA-2 composite correlated with a self-report psychological variable but not detectably with objectively assessed performance. An r of −0.461 corresponds to roughly 21% shared variance, which is substantial for a cross-sectional relationship between two self-report constructs measured in a non-clinical sample. Importantly, the association was not confined to a single MAIA-2 dimension: Attention Regulation, Trusting, Self-Regulation, and Noticing remained inversely related to PSS-10 after FDR correction in the post hoc subscale analysis (Supplementary Table S6). This pattern strengthens the descriptive robustness of the stress association, but it does not establish directionality or mechanism. The finding therefore confirms rather than discovers: it extends an established association to a sport-science student sample while placing it alongside objectively measured performance. The practical implication remains narrow. These data do not establish that interoceptive awareness is modifiable, that changing it would reduce perceived stress, or that the MAIA-2 should be used to select or monitor athletes.

### 4.3. Sex-Related Differences

Men demonstrated markedly higher handgrip strength and CMJ height than women. These differences are consistent with established sex-related variation in body composition, muscle architecture, neuromuscular characteristics, and hormonal milieu that can influence maximal strength and power (Ansdell et al., 2020). Nevertheless, the present study did not directly measure lean mass, muscle cross-sectional area, or endocrine variables. The observed sex coefficients should therefore be interpreted as aggregate associations that may also reflect anthropometry and training exposure. Body mass explained additional variance in the sensitivity models, so not all of the performance difference should be attributed to sex itself.

Women also reported higher perceived stress and state and trait anxiety, and these differences survived FDR correction. The findings identify a group-level psychological pattern in this sample but do not establish a universal sex difference or a biological mechanism. No sex difference emerged for the MAIA-2 composite. Men and women in this cohort reported similar overall interoceptive awareness, despite differing on several psychological and physical outcomes.

### 4.4. Sport Type and State Anxiety

The only sport-type comparison that survived multiplicity correction was higher state anxiety among individual-sport participants. This matches earlier reports that individual-sport athletes experience more anxiety or depressive symptoms than team-sport athletes (Pluhar et al., 2019), and evidence of sport-type differences in competitive anxiety (Kemarat et al., 2022). One plausible explanation is that responsibility for performance outcomes is less distributed in individual sports, while team contexts can provide immediate social support. However, the present categorization combined heterogeneous disciplines and did not measure competition proximity, competitive importance, perfectionism, or perceived social support. The finding should therefore be regarded as provisional rather than as evidence of a sport-type mechanism.

### 4.5. Sleep Quality and Jump Performance

Poorer PSQI scores were modestly associated with lower CMJ height in one adjusted model. This direction is compatible with literature linking sleep disruption with impaired neuromuscular function and athletic performance (Fullagar et al., 2015) and with recent observations in basketball players (Cabarkapa et al., 2024). However, the psychological block as a whole did not significantly improve the CMJ model, and the PSQI coefficient weakened after adjustment for body mass and sport experience. Given the number of secondary analyses, this result should be considered exploratory and should not be interpreted as evidence that PSQI-defined sleep quality causally determines jump performance.

### 4.6. Interpretation of the Indirect-Effect Analyses

The exploratory indirect-effect models (Supplementary Table S3) did not identify statistical pathways from the MAIA-2 composite to physical performance through perceived stress, trait anxiety, or sleep quality. The pattern was particularly informative for perceived stress: interoceptive awareness was related to stress, but stress did not form a stable path to performance after adjustment. These cross-sectional estimates cannot adjudicate causal mediation (Maxwell & Cole, 2007); the appropriate conclusion is that no cross-sectional indirect association was detected, not that psychological mediation has been excluded.

### 4.7. Strengths and Limitations

Several features strengthen the study. Psychological measures were paired with objective field-performance tests spanning strength, power, flexibility, and dynamic balance. Primary findings were re-evaluated using confidence intervals, multiplicity control, Welch tests, heteroscedasticity-robust regression, and post hoc equivalence testing. The investigator-defined 37-item MAIA-2 composite showed a moderate inverse relationship with perceived stress and no detectable relationship with the five physical performance outcomes. Canonical subscale sensitivity analyses, full coefficient reporting, anthropometric/training-history sensitivity models, and Y-Balance exclusion analyses were added during peer review to test the robustness and limits of these inferences.

The limitations are substantial and define the scope of inference. First, the cross-sectional design precludes temporal and causal conclusions, particularly for indirect-effect models. Second, the convenience sample was small, came from a single university, and included sport-science students with heterogeneous competitive backgrounds rather than a clearly defined elite-athlete cohort. Nine participants reported no active sport. Third, the MAIA-2 is principally a multidimensional subscale instrument; the investigator-defined 37-item operational composite should not be treated as a validated unidimensional total score. Two MAIA-2 subscales also showed low internal consistency in this sample, and the post hoc subscale analyses were exploratory and multiplicity-adjusted. Fourth, performance was measured during a single session. Familiarization, inter-rater reliability, recent training load, caffeine and food intake, time of day, motivation, and menstrual-cycle phase were not standardized or modeled. The fixed test order also allows possible order or fatigue effects. Fifth, important determinants of strength and power, particularly body composition and muscle morphology, were not measured; body mass and sport experience provided only partial sensitivity adjustment. Sixth, all psychological variables were self-reported, creating potential common-method and social-desirability bias. Seventh, sport-type categories combined diverse disciplines; competition level was recorded but was missing for two active participants and could not capture competitive proximity or social-support context. Eighth, several normalized Y-Balance values were at the upper end of the observed range. Recalculation confirmed the scores, and exclusion analyses did not alter the inference, but protocol and anthropometric variability cannot be completely excluded. Finally, the study included multiple secondary, exploratory, and post hoc analyses; findings beyond the primary MAIA–performance question should therefore be interpreted conservatively.

### 4.8. Directions for Future Research

Several priorities follow from these results. First, the present battery sampled only brief maximal and capacity-oriented tasks. Testing the task-specificity account directly will require paradigms in which effort must be continuously regulated, such as time-trial, pacing, fatigue-monitoring, or decision-making-under-strain protocols. Second, self-report should be paired with behavioural interoceptive measures, because self-reported awareness and objective accuracy correspond only modestly (Forkmann et al., 2016). Combining MAIA-2 subscales with heartbeat-detection or respiratory tasks would show which level of interoceptive processing, if any, relates to performance. Third, the interoception–stress association should be followed longitudinally and measured with physiological indices such as heart-rate variability or cortisol. This would establish temporal ordering and reduce the common-method variance that two concurrent self-reports inevitably carry. Fourth, sport-type contrasts require larger samples with more complete and granular characterization of competition level, competitive proximity, and perceived social support rather than relying mainly on broad discipline categories. Finally, adequately powered studies are needed: the present sample could reliably detect correlations of |r| ≥ 0.30 only, and detecting smaller but potentially meaningful associations would require substantially larger samples or repeated-measures designs.

## 5. Conclusions

Among sport-science students, self-reported interoceptive awareness was moderately associated with lower perceived stress but was not detectably associated with handgrip strength, countermovement-jump height, flexibility, or dynamic balance. Interoceptive awareness did not significantly increase explained variance in these performance outcomes, and post hoc equivalence testing indicated that four of the five performance correlations were statistically equivalent to zero within a ±0.30 bound set by the study’s statistical sensitivity rather than by an established threshold of practical importance. Exploratory cross-sectional indirect-effect estimates through perceived stress, trait anxiety, and sleep quality were likewise compatible with the null. Canonical MAIA-2 subscale analyses identified several nominal subscale–performance correlations, but none survived FDR correction across 40 tests. These findings are compatible with, but do not directly test, a task-specific account of interoception; because no endurance, pacing, fatigue-regulation, or decision-making task was included, task specificity remains a hypothesis for future work rather than a conclusion supported by the present design. The contribution should therefore be framed narrowly. The inverse association with perceived stress largely confirms existing work, although its presence across several MAIA-2 subscales after multiplicity correction supports its descriptive robustness in this sample. The principal added value is the joint evaluation of psychological state and multiple objective field-performance domains, together with multiplicity control, equivalence testing, and transparent sensitivity analyses that bound the strength of the MAIA–performance association.

This observational study was retrospectively registered at ClinicalTrials.gov (NCT07696234) after completion of data collection. The registration is reported as a study record and not as evidence of prospective preregistration; no intervention was assigned as part of the study.

## Figures and Tables

**Table 1:** Participant characteristics and study-variable distributions (*N* = 85).

Variable	Mean	SD	Range
Age (years)	20.65	2.43	18–30
Height (cm)	171.93	8.40	153.0–195.0
Body mass (kg)	70.11	12.83	44.9–108.5
BMI (kg/m^2^)	23.63	3.33	16.29–32.56
Sport experience (years)	4.11	3.48	0–16
Training sessions/week	2.78	1.82	0–8
Training duration (min/day)	68.41	54.09	0–270
MAIA-2 37-item composite	3.05	0.51	1.11–4.19
PSS-10	17.72	5.65	3–35
STAI-State	43.16	11.61	20–72
STAI-Trait	41.27	7.83	21–59
PSQI	7.48	2.95	0–15
Handgrip strength (kg)	42.36	12.27	21.6–83.0
Countermovement jump (cm)	31.57	9.76	12.17–54.23
Sit-and-reach (cm)	36.36	7.09	11.0–49.5
Y-Balance right (%)	107.22	11.33	83.7–141.1
Y-Balance left (%)	108.31	12.50	81.7–141.5

BMI, body mass index; MAIA-2, Multidimensional Assessment of Interoceptive Awareness, Version 2; PSS-10, Perceived Stress Scale-10; STAI, State-Trait Anxiety Inventory; PSQI, Pittsburgh Sleep Quality Index.

**Table 2:** Pearson associations with the MAIA-2 37-item operational composite.

Outcome	r	95% CI	*p*	FDR q
Perceived stress (PSS-10)	−0.461	−0.614 to −0.275	<0.001	<0.001
State anxiety (STAI-State)	−0.177	−0.376 to 0.038	0.105	0.141
Trait anxiety (STAI-Trait)	−0.210	−0.405 to 0.003	0.053	0.107
Sleep quality (PSQI)	−0.150	−0.352 to 0.065	0.170	0.170
Handgrip strength (kg)	0.000	−0.213 to 0.213	0.999	0.999
Countermovement jump (cm)	0.077	−0.139 to 0.285	0.485	0.961
Sit-and-reach (cm)	0.061	−0.154 to 0.271	0.577	0.961
Y-Balance right (%)	0.136	−0.080 to 0.339	0.215	0.961
Y-Balance left (%)	−0.003	−0.216 to 0.210	0.979	0.999

FDR correction was applied separately to the four psychological associations and the five physical-performance associations. Higher PSQI scores indicate poorer sleep quality.

**Table 3:** Sex comparisons using Welch tests (*N* = 85).

Variable	Men (*n* = 54)	Women (*n* = 31)	Mean Difference (95% CI)	Hedges g	*p*	FDR q
MAIA-2 composite	3.07 ± 0.50	3.01 ± 0.53	0.06 (−0.17 to 0.30)	0.123	0.590	0.590
PSS-10	16.72 ± 6.23	19.45 ± 4.01	−2.73 (−4.94 to −0.52)	−0.489	0.016	0.032
STAI-State	40.11 ± 10.90	48.48 ± 11.04	−8.37 (−13.32 to −3.42)	−0.758	0.001	0.004
STAI-Trait	39.46 ± 7.85	44.42 ± 6.83	−4.96 (−8.20 to −1.71)	−0.655	0.003	0.008
PSQI	7.15 ± 3.10	8.06 ± 2.61	−0.92 (−2.17 to 0.34)	−0.309	0.151	0.251
Handgrip (kg)	49.29 ± 9.53	30.29 ± 4.89	19.00 (15.89 to 22.12)	2.306	<0.001	<0.001
CMJ (cm)	36.26 ± 8.85	23.40 ± 4.42	12.86 (9.99 to 15.73)	1.687	<0.001	<0.001
Sit-and-reach (cm)	36.04 ± 8.13	36.92 ± 4.86	−0.88 (−3.68 to 1.92)	−0.122	0.534	0.590
Y-Balance right (%)	106.65 ± 10.32	108.20 ± 13.03	−1.55 (−7.03 to 3.92)	−0.135	0.572	0.590
Y-Balance left (%)	107.38 ± 11.63	109.93 ± 13.94	−2.55 (−8.48 to 3.39)	−0.202	0.394	0.562

Mean difference = men − women. Negative values indicate higher scores among women. FDR q values were calculated across the 10 comparisons.

**Table 4:** Sport-type comparisons using Welch tests (*N* = 76).

Variable	Team (*n* = 44)	Individual (*n* = 32)	Mean Difference (95% CI)	Hedges g	*p*	FDR q
MAIA-2 composite	3.02 ± 0.48	3.16 ± 0.45	−0.14 (−0.35 to 0.08)	−0.292	0.206	0.616
PSS-10	17.45 ± 5.53	17.66 ± 5.94	−0.20 (−2.88 to 2.48)	−0.035	0.881	0.979
STAI-State	39.20 ± 9.99	48.94 ± 12.00	−9.73 (−14.94 to −4.53)	−0.886	<0.001	0.004
STAI-Trait	39.39 ± 7.87	43.16 ± 7.54	−3.77 (−7.33 to −0.21)	−0.482	0.038	0.191
PSQI	7.25 ± 3.11	7.69 ± 2.46	−0.44 (−1.71 to 0.84)	−0.152	0.496	0.826
Handgrip (kg)	44.22 ± 12.48	41.13 ± 12.57	3.10 (−2.71 to 8.91)	0.245	0.291	0.616
CMJ (cm)	31.87 ± 9.43	31.83 ± 10.08	0.04 (−4.52 to 4.59)	0.004	0.987	0.987
Sit-and-reach (cm)	35.81 ± 7.82	37.53 ± 6.69	−1.71 (−5.04 to 1.61)	−0.230	0.308	0.616
Y-Balance right (%)	108.13 ± 12.00	107.02 ± 11.35	1.12 (−4.27 to 6.51)	0.094	0.681	0.973
Y-Balance left (%)	108.23 ± 12.57	109.05 ± 13.58	−0.81 (−6.92 to 5.30)	−0.062	0.791	0.979

Mean difference = team − individual. Nine participants without an active sport were excluded from these comparisons. FDR q values were calculated across the 10 comparisons.

**Table 5:** Hierarchical regression models for physical performance (*N* = 85).

Outcome	Step 1 R^2^	Step 2 ΔR^2^ (*p*)	Step 3 ΔR^2^ (*p*)	MAIA B (95% CI), *p*	Female B (95% CI), *p*	PSQI B (95% CI), *p*
Handgrip strength	0.562	0.002 (0.539)	0.013 (0.488)	−0.78 (−4.16 to 2.60), 0.648	−18.55 (−22.03 to −15.07), <0.001	−0.22 (−0.93 to 0.49), 0.535
CMJ height	0.414	0.001 (0.699)	0.052 (0.064)	0.25 (−3.66 to 4.15), 0.900	−11.71 (−14.91 to −8.51), <0.001	−0.68 (−1.34 to −0.01), 0.046
Sit-and-reach	0.004	0.004 (0.565)	0.022 (0.626)	1.75 (−1.80 to 5.29), 0.330	0.44 (−2.65 to 3.53), 0.778	0.22 (−0.31 to 0.75), 0.415
Y-Balance right	0.005	0.019 (0.208)	0.044 (0.301)	4.31 (−1.48 to 10.09), 0.142	2.23 (−3.92 to 8.37), 0.473	0.11 (−0.92 to 1.15), 0.828
Y-Balance left	0.011	0.000 (0.995)	0.015 (0.754)	0.32 (−6.91 to 7.55), 0.930	3.27 (−3.59 to 10.12), 0.346	−0.04 (−1.13 to 1.06), 0.947

Step 1: age + sex; Step 2: + MAIA-2 composite; Step 3: + PSS-10 + STAI-Trait + PSQI. Final-model coefficient confidence intervals and *p* values use HC3 robust standard errors. Sex was coded 0 = men, 1 = women. Coefficients for age, PSS-10, and STAI-Trait are omitted here for readability; complete Step 3 coefficients are reported in Supplementary Table S1, and full expanded sensitivity models including body mass and sport experience are reported in Supplementary Table S2.

## Data Availability

De-identified data supporting the findings of this study are available from the corresponding author upon reasonable request and subject to applicable ethical and institutional requirements.

## Supplementary Materials

The following supporting information can be downloaded at: https://www.mdpi.com/article/10.3390/bs16101784/s1; Table S1: Full Step 3 hierarchical regression coefficients for all predictors and five performance outcomes (HC3 robust standard errors); Table S2: Full expanded sensitivity regression coefficients including body mass and sport experience; Table S3: Exploratory age- and sex-adjusted indirect-effect estimates (5000 bootstrap samples); Table S4: Equivalence (TOST) results for MAIA-2–performance correlations; Table S5: Post hoc correlations between the eight canonical MAIA-2 subscales and five performance outcomes with FDR-adjusted q values; Table S6: Post hoc correlations between the eight MAIA-2 subscales and perceived stress with FDR-adjusted q values; Figure S1: Forest plot of exploratory indirect-effect estimates.
