# Supplementary material for: Associations Between Interoceptive Awareness, Psychological State, and Physical Performance in Sport-Science Students: A Cross-Sectional Study
Source: Behav Sci (Basel). 2026 Oct 1;16(10):1784. doi: 10.3390/bs16101784 (PMC13701454; doi:10.3390/bs16101784)
Supplement: Supplementary file 1 [file behavsci-16-01784-s001.zip › behavsci-4527803-supplementary.pdf]

Supplementary Materials

Associations Between Interoceptive Awareness, Psychological State, and Physical Performance in Sport-Science Students: A Cross-Sectional Study

Zhaoqing Li, Emir Mert Koçoğlu, Özgür Eken, Tahir Volkan Aslan, Yuhao Su

Table S1. Full Step 3 hierarchical regression coefficients for all predictors and five performance outcomes (HC3 robust standard errors)

All predictors entered at Step 3 are reported for each of the five physical performance outcomes (N = 85). The MAIA-2 composite is the investigator-defined mean of all 37 appropriately keyed items. Sex was coded 0 = men, 1 = women. Higher PSQI scores indicate poorer sleep quality.

Outcome	Predictor	B	SE (HC3)	95% CI	p
Handgrip strength (kg)	Intercept	56.719	15.316	26.23 to 87.21	<.001
	Age (years)	-.004	.378	-.76 to .75	.992
	Sex (women = 1)	-18.549	1.748	-22.03 to -15.07	<.001
	MAIA-2 composite	-.777	1.697	-4.16 to 2.60	.648
	PSS-10	.203	.209	-.21 to .62	.335
	STAI-Trait	-.172	.168	-.51 to .16	.310
	PSQI	-.222	.357	-.93 to .49	.535
CMJ height (cm)	Intercept	51.124	14.837	21.59 to 80.66	<.001
	Age (years)	-.382	.511	-1.40 to .64	.457
	Sex (women = 1)	-11.711	1.607	-14.91 to -8.51	<.001
	MAIA-2 composite	.247	1.960	-3.66 to 4.15	.900
	PSS-10	.110	.227	-.34 to .56	.627
	STAI-Trait	-.122	.128	-.38 to .13	.341
	PSQI	-.676	.334	-1.34 to -.01	.046
Sit-and-reach (cm)	Intercept	27.268	9.262	8.83 to 45.71	.004
	Age (years)	-.018	.275	-.56 to .53	.948
	Sex (women = 1)	.438	1.552	-2.65 to 3.53	.778
	MAIA-2 composite	1.746	1.782	-1.80 to 5.29	.330
	PSS-10	.134	.152	-.17 to .44	.382
	STAI-Trait	-.001	.120	-.24 to .24	.996
	PSQI	.220	.268	-.31 to .75	.415
Y-Balance right (%)	Intercept	98.235	12.832	72.69 to 123.78	<.001
	Age (years)	.029	.456	-.88 to .94	.950
	Sex (women = 1)	2.226	3.085	-3.92 to 8.37	.473
	MAIA-2 composite	4.306	2.904	-1.48 to 10.09	.142
	PSS-10	.415	.264	-.11 to .94	.120
	STAI-Trait	-.333	.221	-.77 to .11	.135
	PSQI	.113	.518	-.92 to 1.15	.828
Y-Balance left (%)	Intercept	115.527	16.883	81.92 to 149.14	<.001
	Age (years)	-.160	.612	-1.38 to 1.06	.795
	Sex (women = 1)	3.267	3.444	-3.59 to 10.12	.346
	MAIA-2 composite	.320	3.630	-6.91 to 7.55	.930
	PSS-10	.200	.309	-.42 to .82	.520
	STAI-Trait	-.227	.248	-.72 to .27	.365
	PSQI	-.037	.551	-1.13 to 1.06	.947

Table S2. Full expanded sensitivity regression coefficients including body mass and sport experience

Each sensitivity model contains age, sex, the MAIA-2 composite, PSS-10, STAI-Trait, PSQI, body mass, and years of sport experience (N = 85).

Outcome	Predictor	B	SE (HC3)	95% CI	p
Handgrip strength (kg)	Intercept	32.604	14.632	3.46 to 61.75	.029
	Age (years)	-.112	.377	-.86 to .64	.767
	Sex (women = 1)	-14.542	1.928	-18.38 to -10.70	<.001

Outcome	Predictor	B	SE (HC3)	95% CI	p
	MAIA-2 composite	-.673	1.555	-3.77 to 2.42	.666
	PSS-10	.210	.207	-.20 to .62	.315
	STAI-Trait	-.119	.138	-.39 to .16	.390
	PSQI	-.235	.294	-.82 to .35	.427
	Body mass (kg)	.318	.113	.09 to .54	.006
	Sport experience (years)	.028	.253	-.48 to .53	.913
CMJ height (cm)	Intercept	64.108	15.007	34.22 to 94.00	<.001
	Age (years)	-.329	.501	-1.33 to .67	.514
	Sex (women = 1)	-13.524	1.945	-17.40 to -9.65	<.001
	MAIA-2 composite	-.002	1.913	-3.81 to 3.81	.999
	PSS-10	.101	.210	-.32 to .52	.633
	STAI-Trait	-.149	.136	-.42 to .12	.275
	PSQI	-.642	.342	-1.32 to .04	.064
	Body mass (kg)	-.180	.081	-.34 to -.02	.030
	Sport experience (years)	.232	.240	-.25 to .71	.337
Sit-and-reach (cm)	Intercept	36.201	13.736	8.84 to 63.56	.010
	Age (years)	.023	.306	-.59 to .63	.941
	Sex (women = 1)	-1.096	2.092	-5.26 to 3.07	.602
	MAIA-2 composite	1.737	1.879	-2.00 to 5.48	.358
	PSS-10	.132	.151	-.17 to .43	.386
	STAI-Trait	-.020	.123	-.27 to .23	.871
	PSQI	.220	.271	-.32 to .76	.419
	Body mass (kg)	-.116	.103	-.32 to .09	.261
	Sport experience (years)	-.047	.330	-.70 to .61	.888
Y-Balance right (%)	Intercept	112.919	13.743	85.55 to 140.29	<.001
	Age (years)	.086	.420	-.75 to .92	.837
	Sex (women = 1)	.391	3.666	-6.91 to 7.69	.915
	MAIA-2 composite	3.903	2.818	-1.71 to 9.52	.170
	PSS-10	.400	.258	-.11 to .91	.125
	STAI-Trait	-.363	.217	-.80 to .07	.099
	PSQI	.169	.530	-.89 to 1.22	.750
	Body mass (kg)	-.209	.098	-.40 to -.01	.037
	Sport experience (years)	.418	.351	-.28 to 1.12	.237
Y-Balance left (%)	Intercept	129.530	17.405	94.87 to 164.20	<.001
	Age (years)	-.102	.581	-1.26 to 1.05	.861
	Sex (women = 1)	1.297	4.056	-6.78 to 9.38	.750
	MAIA-2 composite	.059	3.536	-6.98 to 7.10	.987
	PSS-10	.190	.311	-.43 to .81	.543
	STAI-Trait	-.256	.251	-.76 to .24	.312
	PSQI	-.001	.575	-1.15 to 1.14	.999
	Body mass (kg)	-.194	.120	-.43 to .05	.111
	Sport experience (years)	.240	.396	-.55 to 1.03	.545

Table S3. Exploratory age- and sex-adjusted indirect-effect estimates (5000 bootstrap samples)

All 95% bootstrap confidence intervals include zero. Because exposure, mediators, and outcomes were measured at a single time point, these estimates are descriptive statistical decompositions and are not interpreted as causal mediation.

Physical outcome	Statistical mediator	Indirect effect	95% bootstrap CI
Handgrip strength	PSS-10	-1.01	-3.58 to 0.74
	STAI-Trait	0.50	-0.68 to 2.35
	PSQI	0.19	-0.52 to 1.34
CMJ height	PSS-10	-0.55	-2.70 to 1.80
	STAI-Trait	0.36	-0.51 to 1.70
	PSQI	0.58	-0.55 to 2.06
Sit-and-reach	PSS-10	-0.67	-2.20 to 1.06
	STAI-Trait	0.00	-0.87 to 1.17
	PSQI	-0.19	-1.26 to 0.23
Y-Balance right	PSS-10	-2.07	-5.06 to 0.74

Physical outcome	Statistical mediator	Indirect effect	95% bootstrap CI
	STAI-Trait	0.97	-0.57 to 3.91
	PSQI	-0.10	-1.69 to 0.83
Y-Balance left	PSS-10	-1.00	-3.98 to 2.50
	STAI-Trait	0.66	-0.83 to 3.45
	PSQI	0.03	-1.54 to 1.26

Table S4. Equivalence (TOST) results for MAIA-2–performance correlations

Two one-sided tests were conducted using symmetric equivalence bounds of ±0.30. The bound was selected during peer review because |r| = .30 was the smallest bivariate correlation detectable with approximately 80% power in this sample; it was not prospectively preregistered. Ninety per cent confidence intervals correspond to the two one-sided tests at α = .05.

Outcome	r	90% CI	TOST p	Equivalent within ±0.30
Handgrip strength (kg)	.000	-.180 to .180	.003	Yes
CMJ height (cm)	.077	-.104 to .253	.018	Yes
Sit-and-reach (cm)	.061	-.120 to .238	.012	Yes
Y-Balance right (%)	.136	-.045 to .308	.059	No
Y-Balance left (%)	-.003	-.182 to .177	.003	Yes

Table S5. Post hoc correlations between the eight canonical MAIA-2 subscales and five performance outcomes with FDR-adjusted q values

The eight canonical MAIA-2 subscales were examined against the five physical performance outcomes. Benjamini–Hochberg FDR adjustment was applied across all 40 subscale–performance correlations. Several unadjusted p values were < .05, but none remained significant after FDR correction.

MAIA-2 subscale	Performance outcome	r	p	FDR q
Noticing	Handgrip strength (kg)	.065	.552	.821
Noticing	CMJ height (cm)	.048	.665	.831
Noticing	Sit-and-reach (cm)	.252	.020	.385
Noticing	Y-Balance right (%)	.237	.029	.385
Noticing	Y-Balance left (%)	.168	.124	.588
Not-Distracting	Handgrip strength (kg)	-.272	.012	.385
Not-Distracting	CMJ height (cm)	-.077	.486	.821
Not-Distracting	Sit-and-reach (cm)	-.087	.430	.821
Not-Distracting	Y-Balance right (%)	-.089	.417	.821
Not-Distracting	Y-Balance left (%)	-.040	.714	.864
Not-Worrying	Handgrip strength (kg)	.094	.392	.821
Not-Worrying	CMJ height (cm)	.136	.213	.588
Not-Worrying	Sit-and-reach (cm)	-.141	.198	.588
Not-Worrying	Y-Balance right (%)	-.074	.498	.821
Not-Worrying	Y-Balance left (%)	-.158	.150	.588
Attention Regulation	Handgrip strength (kg)	.196	.073	.548
Attention Regulation	CMJ height (cm)	.153	.161	.588
Attention Regulation	Sit-and-reach (cm)	.009	.933	.957
Attention Regulation	Y-Balance right (%)	.190	.082	.548
Attention Regulation	Y-Balance left (%)	.074	.500	.821
Emotional Awareness	Handgrip strength (kg)	-.056	.610	.821
Emotional Awareness	CMJ height (cm)	-.069	.531	.821
Emotional Awareness	Sit-and-reach (cm)	.120	.272	.681
Emotional Awareness	Y-Balance right (%)	.220	.043	.426
Emotional Awareness	Y-Balance left (%)	.065	.553	.821
Self-Regulation	Handgrip strength (kg)	-.134	.220	.588
Self-Regulation	CMJ height (cm)	-.008	.944	.957
Self-Regulation	Sit-and-reach (cm)	.060	.583	.821
Self-Regulation	Y-Balance right (%)	.031	.780	.891
Self-Regulation	Y-Balance left (%)	-.048	.663	.831
Body Listening	Handgrip strength (kg)	-.057	.606	.821
Body Listening	CMJ height (cm)	-.006	.957	.957
Body Listening	Sit-and-reach (cm)	.023	.834	.926
Body Listening	Y-Balance right (%)	-.012	.917	.957
Body Listening	Y-Balance left (%)	-.139	.203	.588
Trusting	Handgrip strength (kg)	.149	.173	.588
Trusting	CMJ height (cm)	.155	.158	.588
Trusting	Sit-and-reach (cm)	.055	.616	.821
Trusting	Y-Balance right (%)	.062	.573	.821
Trusting	Y-Balance left (%)	.037	.735	.864

Table S6. Post hoc correlations between the eight MAIA-2 subscales and perceived stress with FDR-adjusted q values

Benjamini–Hochberg FDR adjustment was applied across the eight canonical subscale correlations with PSS-10.

MAIA-2 subscale	r with PSS-10	p	FDR q
Noticing	-.247	.023	.045
Not-Distracting	.117	.286	.286
Not-Worrying	-.180	.099	.133
Attention Regulation	-.557	<.001	<.001
Emotional Awareness	-.166	.130	.149
Self-Regulation	-.274	.011	.030
Body Listening	-.215	.048	.076
Trusting	-.440	<.001	<.001

Figure S1

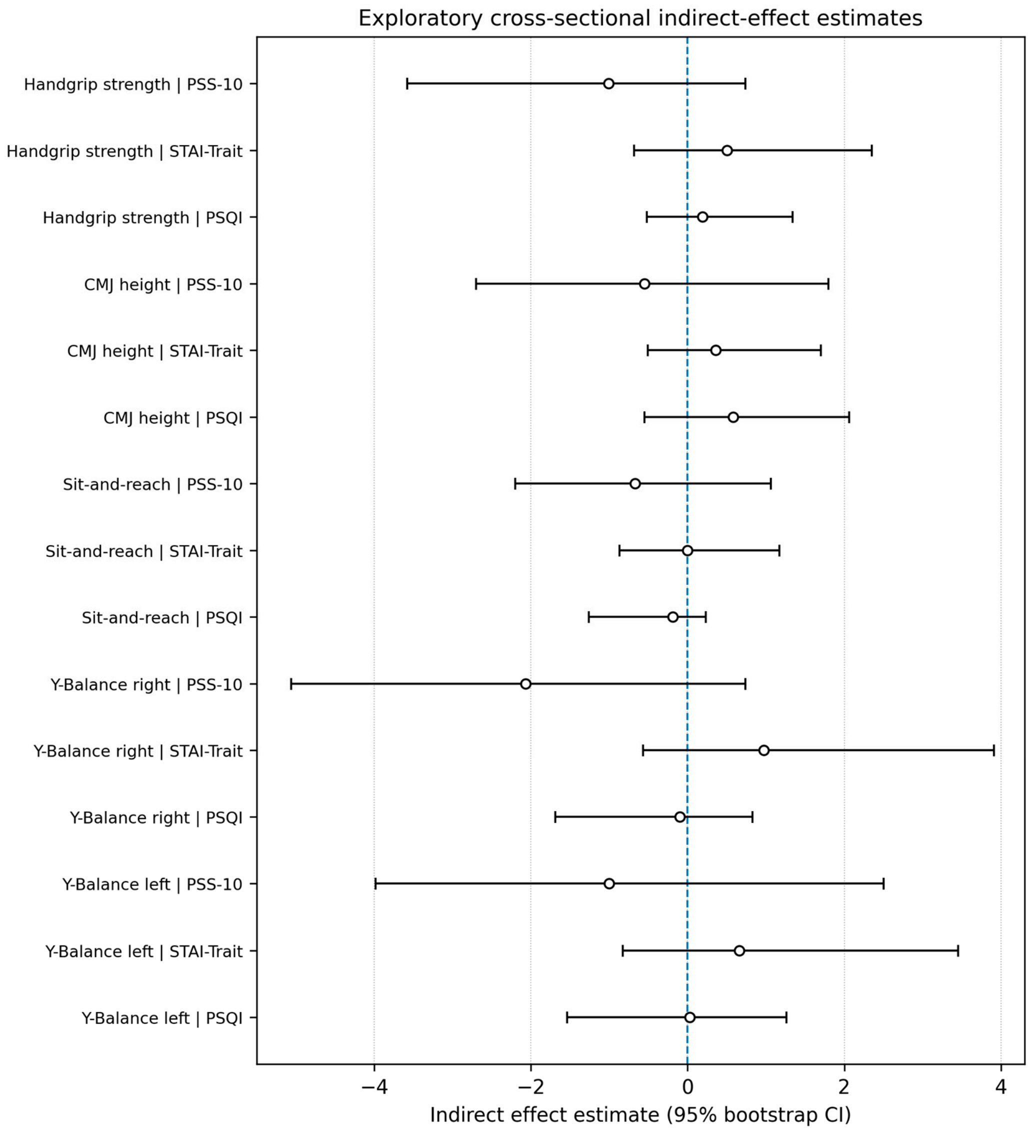

Figure S1. Forest plot of exploratory indirect-effect estimates. Age- and sex-adjusted cross-sectional estimates for five performance outcomes through three statistical mediators (perceived stress, trait anxiety, sleep quality), based on 5000 bootstrap samples. All 95% bootstrap confidence intervals include zero. Because exposure, mediators, and outcomes were measured at a single time point, these estimates are descriptive statistical decompositions and are not interpreted as causal mediation.
